# Supplementary material for: Identification of photoperiod-induced specific miRNAs in the adrenal glands of Sunite sheep (Ovis aries)
Source: Front Vet Sci. 2022 Jul 22;9:888207. doi: 10.3389/fvets.2022.888207 (PMC9354845; doi:10.3389/fvets.2022.888207)
Supplement: Supplementary file 1 [file Data_Sheet_1.ZIP › Supplementary file/miRDeep2 file.zip!/miRDeep2 file/Know_Structure/SP42A3/oar-mir-148a.pdf]

miRBase precursor : oar-mir-148a
Total read count : 1974666
oar-miR-148a read count : 1971620
remaining reads : 3046

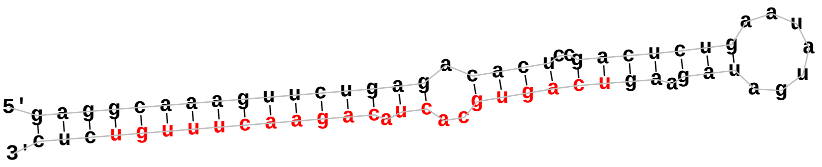

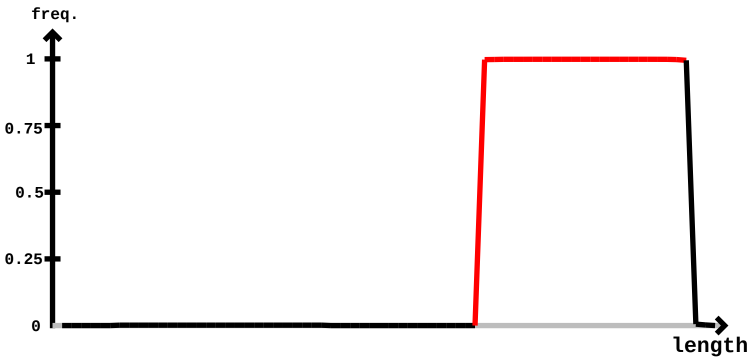


```
                                              oar-miR-148a
5'- gaggcaaaguucugagacacuccgacucugaauaugauagaagucagugcacuacagaacuuugucuc   -3'    exp
    ((((((((((((((((.((((..(((((((.......))).))))))))..)).))))))))))))))    reads      mm         sample
    ....caaaguucugagacacuccga...........................................    2          0          seq
    ....caaaguucugagacacuccgac..........................................    1          0          seq
    ....caaaguucugagacacuccgacu.........................................    62         0          seq
    .....aaaguucugagacacuccg............................................    2          0          seq
    .....aaaguucugagacacuccga...........................................    72         0          seq
    .....aaaguucugagacacuccCa...........................................    1          1          seq
    .....aaaguucugagacacuccgac..........................................    41         0          seq
    .....aaaguucugagacacuccgacu.........................................    2848       0          seq
    .....aaaguucugagacacuccgacuc........................................    4          0          seq
    .....aaaguucugagacacuccgacucu.......................................    7          0          seq
    .....aaaguucugagacacuccgacucug......................................    1          0          seq
    .....aaaguucugagacacuccgacucugaaua..................................    1          0          seq
    ......aaguucugagacacuccgacu.........................................    2          0          seq
    ..................acuccgacucugaauaugauaga...........................    1          0          seq
    ........................................aagucagugcacuacagaacuuu.....    1          0          seq
    .........................................agucagugcacuacagaacuuugu...    1          0          seq
    ..........................................gucagugcacuacagaacuuugu...    75         0          seq
    ...........................................ucagugcacuacagaac........    2          0          seq
    ...........................................ucaguAcacuacagaacu.......    1          1          seq
    ...........................................ucagugcacuacagaacu.......    101        0          seq
    ...........................................uUagugcacuacagaacuu......    2          1          seq
    ...........................................ucagugcacuacagaacuu......    231        0          seq
    ...........................................ucagugcacuacagaacuuu.....    1682       0          seq
    ...........................................uUagugcacuacagaacuuu.....    2          1          seq
    ...........................................uUagugcacuacagaacuuug....    10         1          seq
    ...........................................ucagugcacuacagaacuuug....    5539       0          seq
    ...........................................uUagugcacuacagaacuuugu...    2835       1          seq
    ...........................................ucagugcacuacagaacuuugu...    1947939    0          seq
    ...........................................ucagugcacuacagaacuuuguc..    6121       0          seq
    ...........................................uUagugcacuacagaacuuuguc..    8          1          seq
    ...........................................ucagugcacuacagaacuuugucu.    4519       0          seq
    ...........................................ucagugcacuacagaacuuugucuc    15         0          seq
    ............................................cagugcacuacagaacuuu.....    1          0          seq
    ............................................cagugcacuacagaacuuug....    4          0          seq
    ............................................Uagugcacuacagaacuuugu...    2          1          seq
```

oar-miR-148a

gaggcaaaguucugagacacuccgacucugaauaugauagaagucagugcacuacagaacuuugucuc

....cagugcacuacagaacuuugu...	986	0	seq
....cagugcacuacagaacuuuguc..	10	0	seq
....cagugcacuacagaacuuugucu.	5	0	seq
....agugcacuacagaacuuu.....	2	0	seq
....agugcacuacagaacuuug....	8	0	seq
....agugcacuacagaacuuugu...	1483	0	seq
....agugcacuacagaacuuuguc..	5	0	seq
....agugcacuacagaacuuugucu.	8	0	seq
....gugcacuacagaacuuugu...	5	0	seq
....ugcacuacagaacuuugu...	2	0	seq
....gcaUuacagaacuuugu...	1	1	seq
....gcacuacagaacuuugu...	14	0	seq
....gAacuacagaacuuugu...	1	1	seq
